# Viable and Heat-Killed Probiotic Strains Improve Oral Immunity by Elevating the IgA Concentration in the Oral Mucosa

**DOI:** 10.1007/s00284-021-02569-8

**Published:** 2021-08-03

**Authors:** Wen-Yang Lin, Yi-Wei Kuo, Ching-Wei Chen, Yu-Fen Huang, Chen-Hung Hsu, Jia-Hung Lin, Cheng-Ruei Liu, Jui-Fen Chen, Ko-Chiang Hsia, Hsieh-Hsun Ho

**Affiliations:** Department of Research and Design, Bioflag Biotech Co., Ltd., 4F.C2, No. 17, Guoji Rd, Xinshi Dist, Tainan City, 744 Taiwan

## Abstract

Oral-nasal mucosal immunity plays a crucial role in protecting the body against bacterial and viral invasion. Safe probiotic products have been used to enhance human immunity and oral health. In this study, we verified the beneficial effects of mixed viable probiotic tablets, consisting of *Lactobacillus salivarius* subsp. *salicinius* AP-32, *Bifidobacterium animalis* subsp. *lactis* CP-9, and *Lactobacillus paracasei* ET-66, and heat-killed probiotic tablets, consisting of *L. salivarius* subsp. *salicinius* AP-32 and *L. paracasei* ET-66, on oral immunity among 45 healthy participants. Participants were randomly divided into viable probiotic, heat-killed probiotic, and placebo groups. The administration of treatment lasted for 4 weeks. Saliva samples were collected at Weeks 0, 2, 4, and 6, and *Lactobacillus*, *Bifidobacterium* and *Streptococcus mutans* populations and IgA concentration were measured. IgA concentrations, levels of TGF-beta and IL-10 in PBMCs cells were quantified by ELISA method. Results showed that salivary IgA levels were significantly increased on administration of both the viable (119.30 ± 12.63%, ***P < 0.001) and heat-killed (116.78 ± 12.28%, ***P < 0.001) probiotics for 4 weeks. Among three probiotic strains, AP-32 would effectively increase the levels of TGF-beta and IL-10 in PBMCs. The oral pathogen *Streptococcus mutans* was significantly reduced on viable probiotic tablet administration (49.60 ± 31.01%, ***P < 0.001). The in vitro antibacterial test confirmed that viable probiotics effectively limited the survival rate of oral pathogens. Thus, this clinical pilot study demonstrated that oral probiotic tablets both in viable form or heat-killed form could exert beneficial effects on oral immunity via IL-10, TGB-beta mediated IgA secretion. The effective dosage of viable probiotic content in the oral tablet was 10^9^ CFUs/g and the heat-killed oral tablet was 1 × 10^10^ cells/g.

## Introduction

The oral-nasal mucosa, the gateway for contacting inhaled antigens, is the frontline defense of the human immune system [1, 2]. Usually, pathogenic viruses or bacteria invade mucosal surfaces of the oral-nasal cavity by fighting against the local immune system and the healthy microbiota [3]. Inflammation and symptoms, including cough, sore throat, runny nose, and fever, develop due to the immune responses against the invasive pathogenic viruses and bacteria [4].

Additionally, immunoglobulin A (IgA) is the main immune factor in saliva and regulates homeostasis of the oral microbiota [5]. Several studies have reported the ability of secretory IgA to protect the body from infection by viral pathogens, including respiratory syncytial virus [6], rotavirus [7], and influenza virus [7]. Secretory IgA is functioned to protect mucosal epithelia. Nicolas Millet et al. discovered that mucosal IgA can prevent commensal *Candida albicans* dysbiosis in the oral cavity [8]. It also prevents aggregation of the oral pathogen *Streptococcus mutans*, which causes dental plaque [9].

Besides, researchers discovered that the oral microbiota and its metabolites may locally influence immunity at the oral barrier [10]. Previous studies demonstrated the effectiveness of probiotics on the elevating the IgA concentration in the oral mucosa [11]. Ericson et al. demonstrated that chewing gum contained *Lactobacillus reuteri* participated in the adaptive immune response of healthy subjects by increasing the salivary IgA levels [12]. In human, the immune cytokines TGF-beta and IL-10 are correlated to the production of IgA [13].

However, whether oral microbiota elevated secretory IgA level through cytokines TGF-beta and IL-10 are still unclear.

Furthermore, the oral pathogens including *S. mutans*, *P. gingivalis*, *F. nucleatum* subsp*. polymorphum*, and *A. actinomycetemcomitans* showed high correlation with periodontal disease and bad breath [14]. Probiotic product could be a potential alternative therapy to periodontitis by inhibiting oral pathogens *Fusobacterium nucleatum,* but *Porphyromonas gingivalis* and *Aggregatibacter actinomycetemcomitans* showed no significant decrease [15]. Probiotic supplementation can also prevent pathogenic infections [16]. It has been reported that key species of the microbiota limited the growth of pathogenic *Streptococcus pneumonia* [17]. Thus, maintaining a balanced microbial ecosystem via the uptake of probiotic strains may be an alternative way to regulate oral immunity and enhance oral health.

Previous findings on the inhibition of oral pathogens by probiotic strains have revealed that *Lactobacillus salivarius* subsp. *salicinius* AP-32, *Bifidobacterium animalis* subsp. *lactis* CP-9, and *Lactobacillus paracasei* ET-66 exert excellent antibacterial effects in vitro; however, not by the mechanism of increasing H_2_O_2_ levels [18]. The major strategies for probiotic strains to limit oral pathogenic growth were co-aggregating pathogens in oral, producing biosurfactants, to change oral environment, generating antimicrobial substances [19]. Additionally, an in vitro study showed that *L. salicinius* AP-32 mediated inflammatory cytokine [interleukin (IL)-8, IL-10] expression in human epithelial cells [20].

Therefore, in this study, we verified whether mixed viable probiotic tablets, consisting of *L. salivarius* subsp. *salicinius* AP-32, *B. animalis* subsp. *lactis* CP-9, and *L. paracasei* ET-66, and heat-killed probiotic tablets, consisting of *L. salivarius* subsp. *salicinius* AP-32 and *L. paracasei* ET-66, improved immune function via TGF-beta, IL-10 or elevated the level of salivary IgA concentration. The anti-bacterial function of probiotic product uptake in the oral cavity was tested.

## Material and Methods

### Participants

In total, 45 healthy participants aged 20–40 years were recruited and randomly divided into three subgroups: the viable probiotic oral tablet, heat-killed probiotic oral tablet, and placebo groups. Each group consisted of 15 participants. Participants with smoking and betel nut eating habits were excluded. Oral tablets were blindly assigned to the participants. All participants took the oral tablets thrice a day (morning, noontime, and evening) for 4 weeks. Saliva samples (2 mL) were collected to measure IgA concentration and microorganism populations at Weeks 0, 2, 4, and 6. Informed consent was obtained from all participants included in this study. The protocol was approved by the Institutional Review Board of Chung Shan Medical University, Taiwan (CS19052).

### Viable Probiotic Oral Tablets

The viable probiotic oral tablet (1 g) contained three viable probiotic strains, *L. salivarius* subsp. *salicinius* AP-32, *B. animalis* subsp. *lactis* CP-9, and *L. paracasei* ET-66. We mixed 0.01 g of 10^11^ colony formation units (CFUs) single probiotic in oral tablet. Each single probiotic strain was measured around 0.33 * 10^9^ CFUs/g. The total probiotic content in the oral tablet was 10^9^ colony CFUs/g. The active dosage of probiotic oral tablet were following previous published works [18]. All probiotic strains were obtained from the laboratory of Bioflag Biotech Co., Ltd. (Tainan, Taiwan). *L. salivarius* subsp. *salicinius* AP-32 was isolated from healthy human gut and deposited in Food Industry Research and Development Institute, Taiwan (ID: BCRC 910437) and in Wuhan university, China (ID: CCTCC-M2011127); *B. animalis* subsp. *lactis* CP-9 was isolated from healthy human breast milk and deposited in Food Industry Research and Development Institute, Taiwan (ID: BCRC 910645) and in Wuhan university (ID: CCTCC-M2014588); *L. paracasei* ET-66 was isolated from healthy human breast milk and deposited in Food Industry Research and Development Institute, Taiwan (ID: BCRC 910753) and in China General Microbiological Culture Collection Center, Beijing, China (ID: CGMCC-13514).

*Lactobacillus. salivarius* subsp. *salicinius* AP-32 and *L. paracasei* ET-66 were cultured on de Man, Rogosa, and Sharpe (MRS) agar plates (110,660, Merck, Darmstadt, Germany) and incubated under facultative anaerobic conditions at 37 ℃ for 48 h. *B. animalis* subsp. *lactis* CP-9 was cultured on MRS agar supplemented with 0.05% cysteine and anaerobically incubated at 37 ℃ for 48 h. Counting colonies was performed for quantifying bacterial CFU after culturing probiotic strains [18].

### Heat-killed Probiotic Oral Tablets

The heat-killed probiotic oral tablets (1 g) consisted of inactivated *L. salivarius* subsp. *salicinius* AP-32 and *L. paracasei* ET-66. The viable strains were cultured and incubated, as previously described. Then, AP-32 and ET-66 were inactivated by incubating the viable strains in MRS media at 100 ℃ for 1 h. The probiotic content in the oral tablet was 1 × 10^10^ cells/g. The active dosage of heat-killed probiotic oral tablet were following previous published in vitro testing [11]. All experimental procedures were performed in the laboratory of Bioflag Biotech Co., Ltd. (Tainan, Taiwan).

### Measurement of the *Lactobacillus* and *Bifidobacterium* Populations in Saliva Samples

Saliva samples (2 mL) were collected from each participant at Weeks 0, 2, 4, and 6. The saliva samples (100 µL) were serially diluted and added to MRS plates. To measure *Lactobacillus* populations, *Lactobacillus* was cultured and incubated on the MRS agar plates under facultative anaerobic conditions at 37 °C for 48 h. To measure *Bifidobacterium* populations, *Bifidobacterium* was cultured and incubated anaerobically on MRS agar supplemented with 0.05% cysteine at 37 °C for 48 h. Counting colonies was performed for quantifying bacterial CFU after culturing probiotic strains. The *Lactobacillus* and *Bifidobacterium* population rates were normalized to the probiotic population number at Week 0. The population rate of *Lactobacillus* and *Bifidobacterium* collected at Week 0 was considered 100%. The experimental method followed previous published works [18].

### Measurement of the IgA Concentration in the Oral Mucosa

Saliva samples (2 mL) were collected from each participant at Weeks 0, 2, 4, and 6 and maintained at − 20 ℃. The IgA Human Uncoated ELISA Kit (Catalog # 88-50600-88; Invitrogen, Thermo Fisher Scientific, San Jose, CA, USA) was used to analyze the IgA concentration. Firstly, we coated anti-human IgA antibodies on 96-well plate. Salivary IgA present in the sample or standard were added to the microwell, then HRP-conjugated anti-human IgA antibody was added at room temperature for 1 h. Pipetting TMB Substrate to each well for 30 min. Finally, the IgA concertation was measured on a spectro-photometer at 450 nm. All experimental procedure was performed in triplicate, according to manufacturer’s instructions. The concentration rate of IgA collected at Week 0 was considered 100%. The experimental method followed previous published works [18].

### Measurement of the TGF-Beta and IL-10 Concentration in PBMC

The testing of probiotic induced elevation of TGF-beta and IL-10 level was following the previous study [20]. The peripheral blood mononuclear cells (PBMCs) were obtained from blood samples of healthy donors (Tainan Blood Center, Taiwan Blood Services Foundation). Centrifuging whole blood through a Ficoll-Hypaque gradient (Pharmacia, Sweden), and the light-density fraction from the 42.5%-50% interface was eluted. Seeding 4 * 10^5^ cell extracted PBMCs cells in 100 µl RPMI-1640 with 1% FBS (Sigma Chemical Co., St. Louis, MO, USA) to each well of 96 well plate. Then adding 5 * 10^5^ cfu/20 µl probiotic treatment to each PBMCs seeded well (cells: probiotic strains = 1:10). The positive controls were adding 10 µg/ml Phytohaemagglutinin (PHA) (Sigma-Aldrich, Munich, Germany) to PBMCs seeded wells for the production of TGF-β, IL-10 respectively [21]. Co-culturing probiotics with PBMCs for 48 h. Measuring supernatant containing cytokines IL-10 and TGF-β by enzyme-linked immunosorbent assay (ELISA) analysis (Thermo Scientific, Carlsbad, CA, USA). All experiments were measured and followed the commercial protocol at least in triplicate.

### Evaluation of the Antibacterial Function in Oral Cavity

*Streptococcus mutans* is a common oral health threat, which caused dental caries by forming plague or biofilms [22]. *Streptococcus mutans* samples was collected from the swabs, by which we collected the *S. mutans* on supragingival part, gingivae and oral mucosa, at Weeks 0, 2, 4, and 6. *S. mutans* was maintained in tryptic soy broth (TSB, 5 mL) supplemented with 50% glycerol. The *S. mutans* medium was serially diluted and added to the Mitis Salivarius-Bacitracin Agar (MSBA, 0.2 U/mL) plate, which was incubated at 37 ℃ for 2 d. Finally, the number of *S. mutans* colonies in each participant sample was counted to calculate pathogenic survival rates; the survival rate of *S. mutans* collected at Week 0 was considered 100%. The experimental method followed previous published works [18].

### In Vitro Antibacterial Activity of the Oral Tablets

Oral tablets (1 g) that contained viable (AP-32, CP-9, and ET-66; > 10^9^ CFUs) and heat-killed (AP-32 and ET-66, > 10^10^ CFUs) probiotic strains were dissolved in TSB and brain heart infusion medium, respectively, to obtain a final concentration of 0.1 g/mL. Only the medium was used for the blank group, whereas tablets (1 g) without any probiotic components were dissolved in the respective media for the placebo group. Then, oral pathogens (10^6^ CFUs) were introduced into the tablet solutions and co-incubated at 37 ℃ for 2 (*S. mutans*) or 3 (*Porphyromonas gingivalis*, *Fusobacterium nucleatum* subsp*. polymorphum*, and *Aggregatibacter actinomycetemcomitans*) d. Pathogens were obtained from the BCRC. The oral pathogen inhibition rate (%) was calculated as follows: (CFU_blank control_–CFU_experimental group_)/CFU_blank control_. The experimental method followed previous published testing [11].

### Statistical Analysis

GraphPad Prism software was used for statistical analysis of two-tailed t-test results. Data were presented as the mean ± standard deviation (SD) or the mean of the results of two or three independent experiments. Statistical differences were considered significant at *P* value < 0.05.

## Results

### Probiotic Tablets Increased the *Bifidobacterium* Population in the Oral Cavity

**Fig. 1:**
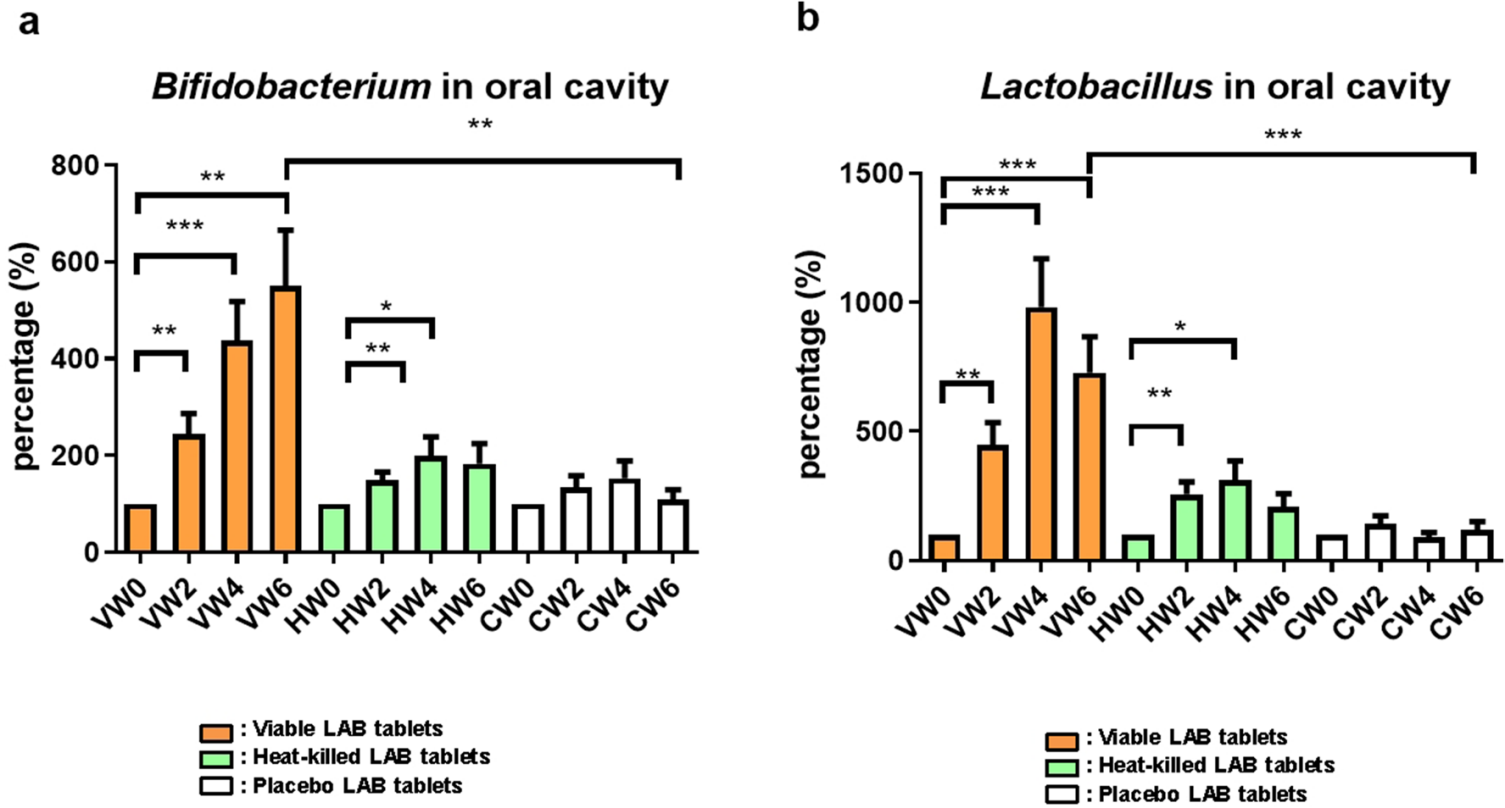
**a** Measurement of *Bifidobacterium* populations on the oral mucosal surfaces. *V* viable probiotic tablets; *H* heat-killed inactivated probiotic tablets; *C* placebo tablets (control); and *LAB* lactic acid bacteria. Collection and analysis of salivary samples at Weeks 0, 2, 4, and 6 were performed. Data were presented as the mean ± standard deviation (SD). Two-tailed *t*-test was used to analyzed statistical difference. Statistical significances were marked with *P < 0.05, **P < 0.01, ***P < 0.001. **b** Measurement of *Lactobacillus* populations on the oral mucosal surfaces. *V* viable probiotic tablets; *H* heat-killed inactivated probiotic tablets; *C* placebo tablets (control); and *LAB* lactic acid bacteria. Collection and analysis of salivary samples at Weeks 0, 2, 4, and 6 were performed. Data were presented as the mean ± standard deviation (SD). Two-tailed *t*-test was used to analyzed statistical difference. Statistical significances were marked with *P < 0.05, **P < 0.01, ***P < 0.001

The *Bifidobacterium* population was stably elevated in the oral cavity after probiotic tablet administration. The bacterial populations after treatments were normalized by bacterial populations before treatments (Population rate % = post-treatment CFU/pre-treatment CFU). In the viable probiotic group, the population rate significantly increased to 244.14 ± 164.96% at Week 2 (**P < 0.01, Fig. 1a) and 438.41 ± 308.58 at Week 4 (***P < 0.001), compared to Week 0. The *Bifidobacterium* population rate remained high at Week 6 (no administration of viable probiotic tablets for 2 weeks after Week 4, 550.16 ± 448.19%, ***P < 0.001), compared to Week 0. The *Bifidobacterium* population number was significantly different between the viable probiotic and placebo groups at Week 6 (**P < 0.01).

In the heat-killed probiotic group, the *Bifidobacterium* population was increased at Weeks 2 (149.92 ± 60.47%, **P < 0.01) and 4 (199.87 ± 148.64%, *P < 0.05), but was decreased at Week 6 after administration of the heat-killed probiotic tablets was stopped for 2 weeks (182.98 ± 163.73%, Fig. 1a).

### Probiotic Tablets Increased the *Lactobacillus* Population in the Oral Cavity

The change in the oral *Lactobacillus* population due to probiotic tablet administration revealed a pattern similar to that followed by the change in the oral *Bifidobacterium* population*.* The bacterial populations after treatments were normalized by bacterial populations before treatments. In the viable probiotic group, the *Lactobacillus* population rate significantly increased to 447.74 ± 337.89% at Week 2 (**P < 0.01, Fig. 1b) and 982.27 ± 726.66% at Week 4 (***P < 0.001), compared to Week 0. The *Lactobacillus* population rate was higher at Week 6 (no administration of viable probiotic tablets for 2 weeks after Week 4, 727.57 ± 539.76%, ***P < 0.001) than Week 0. The *Lactobacillus* population number was significant different between the viable probiotic and placebo groups at Week 6 (***P < 0.001). In the heat-killed probiotic group, the *Lactobacillus* population was increased at Weeks 2 (256.66 ± 183.78%, **P < 0.01) and 4 (312.85 ± 279.71%, *P < 0.05). Additionally, the *Lactobacillus* number remained but showed no significant difference at Week 6 after administration of heat-killed probiotics was stopped for 2 weeks (208.00 ± 199.50%, Fig. 1b**)**.

### Probiotic Tablets Containing AP-32, ET-66, and CP-9 Significantly Elevated the IgA Concentration in the Oral Mucosa

**Fig. 2:**
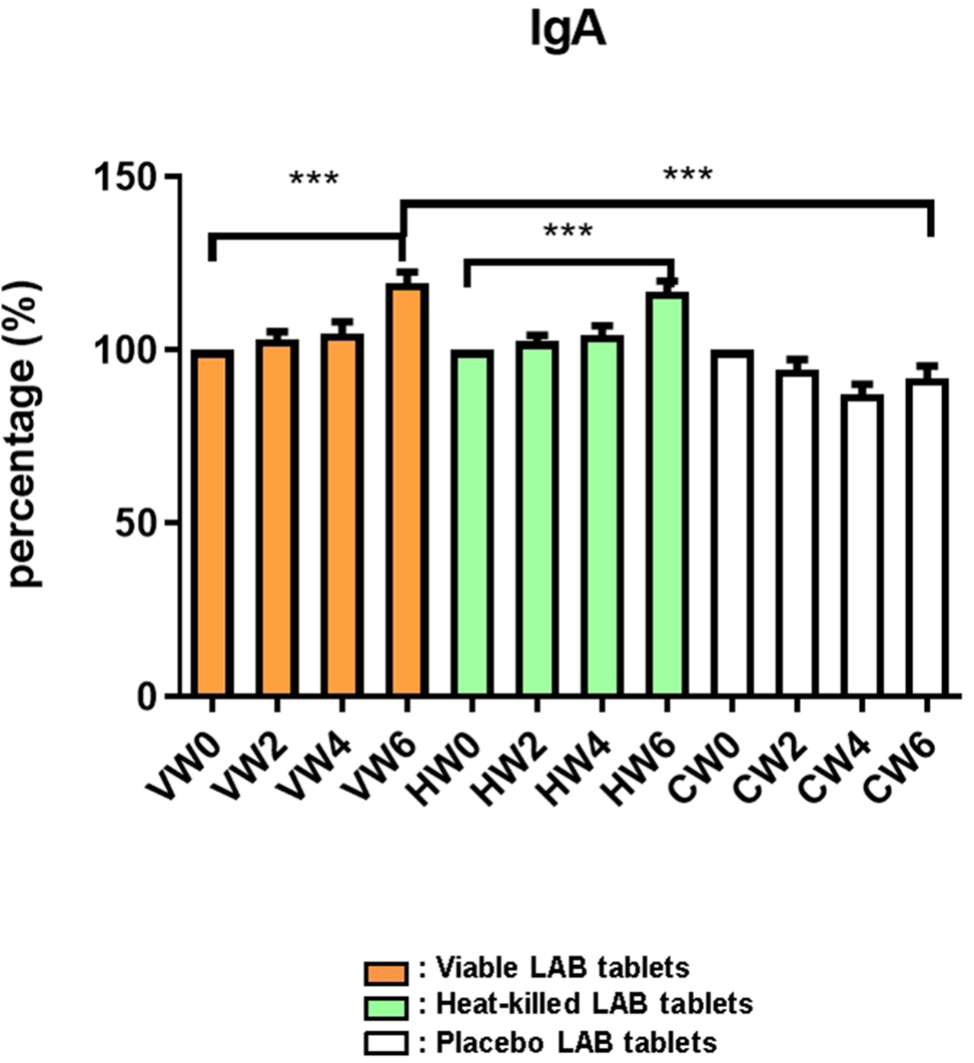
Immunoglobulin A (IgA) concentration in the saliva samples. *V* viable probiotic tablets; *H* heat-killed inactivated probiotic tablets; *C* placebo tablets (control); and *LAB* lactic acid bacteria. Collection and analysis of salivary samples at Weeks 0, 2, 4, and 6 were performed. Data were presented as the mean ± standard deviation (SD). Two-tailed *t*-test was used to analyzed statistical difference. Statistical significances were marked with *P < 0.05, **P < 0.01, ***P < 0.001

As probiotic tablet (viable or heat-killed) consumption has been proven to elevate *Bifidobacterium* and *Lactobacillus* levels in the oral cavity*,* we further analyzed the salivary IgA concentration changes, which occurred due to the uptake of probiotic products. The IgA levels after treatments were normalized by IgA levels before treatments. In the viable probiotic group, the salivary IgA concentration slightly increased to 103.24 ± 8.10% at Week 2 (Fig. 2) and 104.81 ± 13.26 at Week 4, but without statistically significant differences. However, the salivary IgA concentration in the viable probiotic group, compared to in the placebo group, was significantly increased to 119.30 ± 12.63% at Week 6 (no administration of viable probiotic tablets for 2 weeks after Week 4), compared to Week 0 (***P < 0.001). Furthermore, in the heat-killed probiotic group, the salivary IgA concentration was slightly increased at Weeks 2 (102.51 ± 7.20%) and 4 (104.35 ± 10.27%). The saliva IgA concentration was increased at Week 6 (116.78 ± 12.28%, ***P < 0.001, Fig. 2), compared to Week 0.

### Viable Strains Increased IL-10 and TGF-Beta Levels in PBMCs

**Fig. 3:**
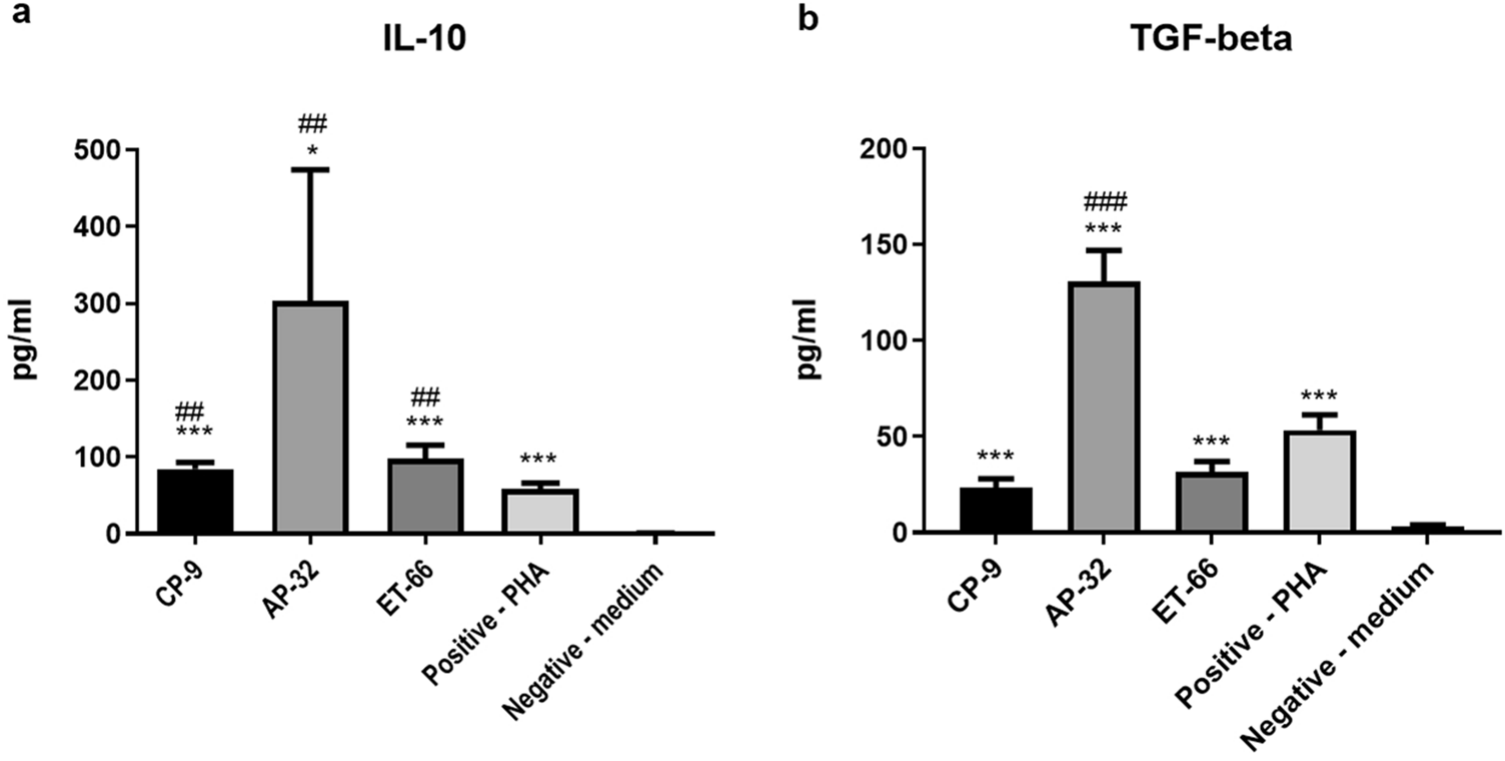
Viable strains increased IL-10, TGF-beta, levels in PBMCs. The level of **a** IL-10 and **b** TGF-beta in PBMCs were tested by treating probiotic strains. Adding 5 * 10^5^ cfu/20 µl viable strains of CP-9, AP-32 and ET-66 to PMBCs 10 µg/ml. Phytohaemagglutinin (PHA) was used as positive control. The negative control was PBMCs treated with RMPI-1640 medium only. Triplicate tests were performed. Data were presented as the mean ± standard deviation (SD). Two-tailed *t*-test was used to analyzed statistical difference. Statistical significances were marked with *P* < 0.05, *P* < 0.01, *P* < 0.001. Symbol *indicated significant difference by comparing to the medium treated control; symbol #indicated significant difference by comparing to the PHA control

The positive control of 10 µg/ml Phytohaemagglutinin (PHA) was successfully elevated the levels of IL-10 (58.2 ± 7.9 pg/ml, ***P < 0.001, Fig. 3a) and TGF-β (53.4 ± 8 pg/ml, ***P < 0.001, Fig. 3b) in PBMCs by comparing to the negative control (PBMCs treated with medium only). Viable strains of CP-9, AP-32 and ET-66 significantly increased the levels of IL-10 in PBMCs to 84.2 ± 9 pg/ml (***P < 0.001, ##*P* < 0.01), 303.1 ± 170.9 pg/ml (*P < 0.05, ##*P* < 0.01) and 98.2 ± 17.5 pg/ml (***P < 0.01, ##*P* < 0.01), respectively. Symbol *indicated significant difference by comparing to the medium treated control; symbol #indicated significant difference by comparing to the PHA control (Fig. 3a).

Besides, viable strains of CP-9, AP-32 and ET-66 significantly increased the levels of TGF-beta in PBMCs to 23.2 ± 4.7 pg/ml (***P < 0.001), 130.9 ± 16 pg/ml (***P < 0.001, ###*P* < 0.001) and 31.4 ± 5.5 pg/ml (***P < 0.01), respectively (Fig. 3b).

### Probiotic Tablets Containing AP-32, ET-66, and CP-9 Prohibited the *S. mutans* Population in the Saliva

**Fig. 4:**
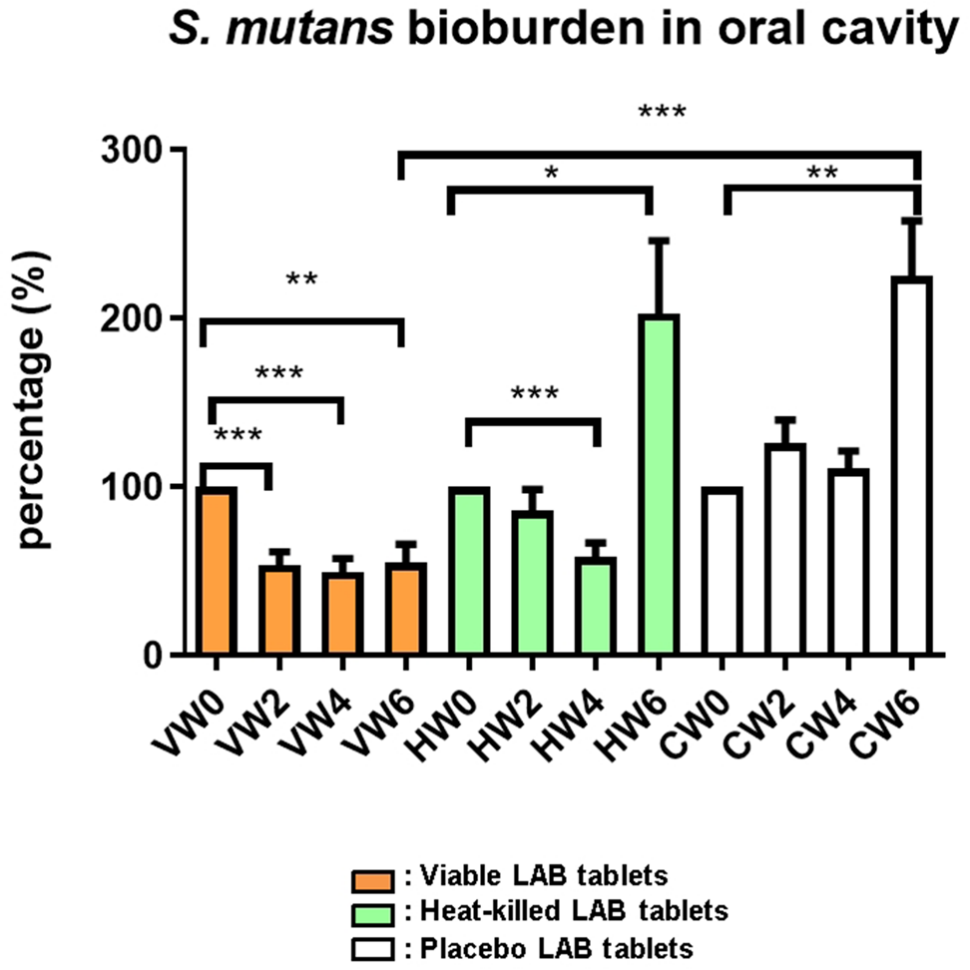
Measurement of pathogenic *Streptococcus mutans* populations on the oral mucosal surfaces. *V* viable probiotic tablets; *H* heat-killed inactivated probiotic tablets; *C* placebo tablets (control); and *LAB* lactic acid bacteria. Collection and analysis of salivary samples at Weeks 0, 2, 4, and 6 were performed. Data were presented as the mean ± standard deviation (SD). Two-tailed *t*-test was used to analyzed statistical difference. Statistical significances were marked with *P < 0.05, **P < 0.01, ***P < 0.001

Elevated *Bifidobacterium* and *Lactobacillus* populations and increased salivary IgA concentration might contribute to the fight against oral pathogenic microorganisms. In the subsequent experiment, we tested the alteration in the oral pathogen *S. mutans* population due to probiotic intervention. The bacterial populations after treatments were normalized by bacterial populations before treatments. In the viable probiotic group, the *S. mutans* population was significantly reduced at Weeks 2 (53.55 ± 31.60%, ***P < 0.001), 4 (49.60 ± 31.01%, ***P < 0.001), and 6 (55.19 ± 42.72%, **P < 0.01, Fig. 4). At week 6, *S. mutans* was significantly inhibited in the viable probiotic group (55.19 ± 42.72%, ***P < 0.001), compared to in the placebo group. Furthermore, in the heat-killed probiotic group, the *S. mutans* population was slightly decreased at Week 2 (85.81 ± 49.87%) and significantly reduced at Week 4 (58.35 ± 33.17%, ***P < 0.001). The *S. mutans* population level in the heat-killed probiotic group (202.81 ± 167.99%) reverted to that in the placebo group (225.05 ± 127.01%) at Week 6 (no administration of heat-killed probiotic tablets for 2 weeks from Week 4) (Fig. 4).

### Confirmation of the Ability of Probiotic Tablets to Inhibit Oral Pathogen Growth by an In Vitro Test

**Fig. 5:**
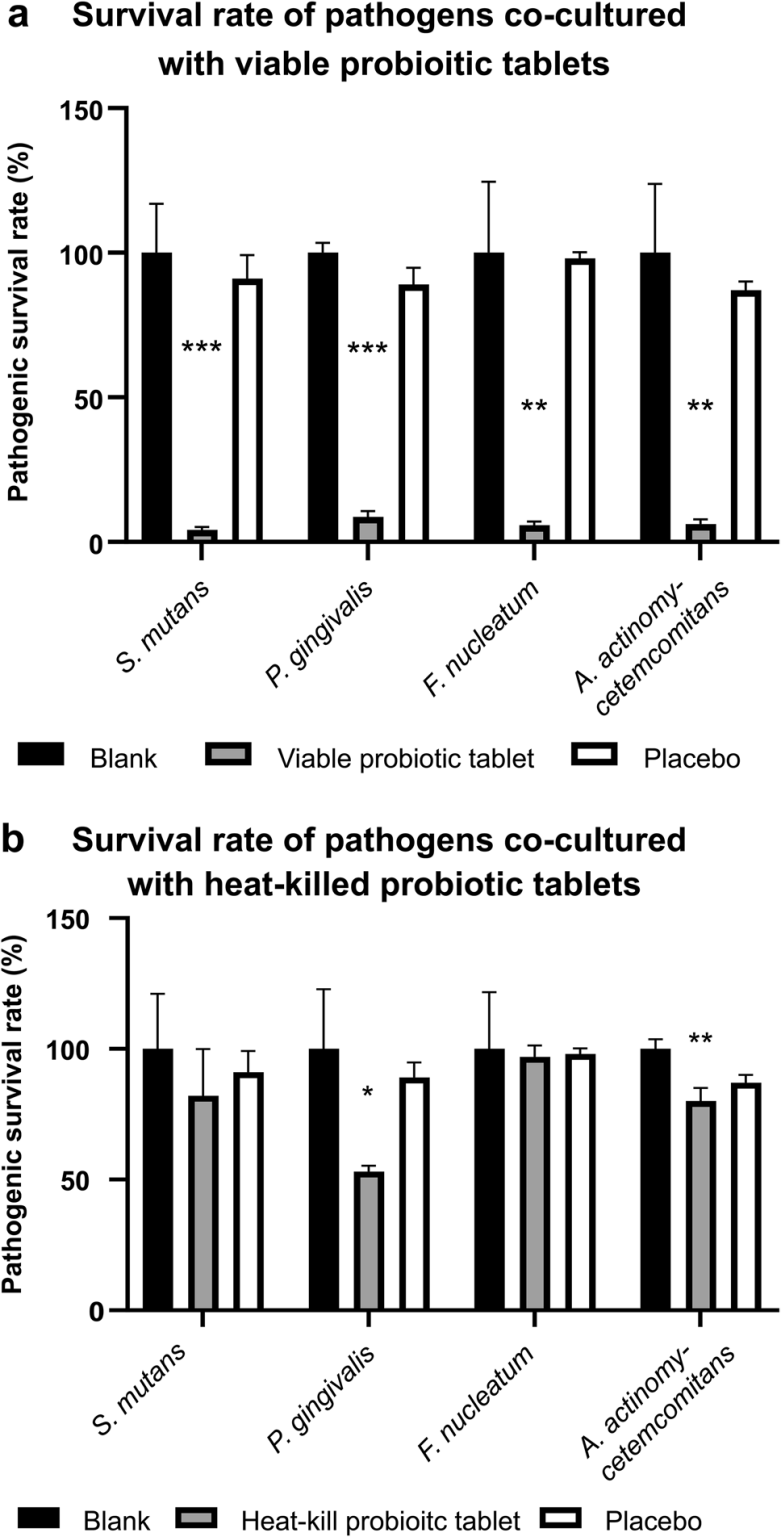
Confirmation of the ability of probiotic tablets to inhibit oral pathogen growth by an in vitro test. The ability of **a** viable and **b** heat-killed probiotic tablets to inhibit the survival rate of oral pathogens, including *Streptococcus mutans, Porphyromonas gingivalis*, *Fusobacterium nucleatum*, and *Aggregatibacter actinomycetemcomitans*, was validated. Blank group, medium only; placebo group, tablets (1 g) without any probiotic components. Triplicate tests were performed. Data were presented as the mean ± standard deviation (SD). Two-tailed *t*-test was used to analyzed statistical difference. Statistical significances were marked with *P < 0.05, **P < 0.01, ***P < 0.001

The in vitro study revealed that viable probiotic tablets significantly inhibited the survival rate of oral pathogens (Fig. 5a). The bacterial populations after treatments were normalized by bacterial populations before treatments. The survival rate of *S. mutans*, *P. gingivalis*, *F. nucleatum*, and *A. actinomycetemcomitans* in the viable probiotic group, compared to in the placebo group, was decreased to 4.23% (***P < 0.001, placebo = 91%), 8.64% (***P < 0.001, placebo = 89%), 5.77% (**P < 0.01, placebo = 98%), and 6.23% (**P < 0.01, placebo = 87%), respectively. However, the heat-killed probiotic tablet exerted weaker antibacterial effects than the viable probiotic tablet (Fig. 5b). The survival rate of *S. mutans, P. gingivalis*, *F. nucleatum*, and *A. actinomycetemcomitans* in the heat-killed probiotic group, compared to in the placebo group, was slightly decreased without statistical significance (82%, placebo = 91%), significantly reduced to 53% (*P < 0.05, placebo = 89%), not inhibited (96.88%, placebo = 98%), and significantly reduced to 80% (**P < 0.01, placebo = 87%), respectively.

## Discussion

Comparing to the placebo group, the oral populations of *Lactobacillus* and *Bifidobacterium* would significantly increase by feeding viable probiotic tablet or heat-killed probiotic tablet for 4 weeks. Additionally, the *Lactobacillus* and *Bifidobacterium* number remained significant high level for 2 weeks after suspension the consumption of viable probiotics consumption (Fig. 1a and b). Similarly, Camila Casuccio Almeida et al. demonstrated that four‐week administration of a probiotic treatment could persist ameliorate symptoms in lactose‐intolerant patients for at least 3 months after stopping probiotic consumption [23]. However, persisted effect of probiotic in long-term treatment should be evaluated in the future.

Additionally, salivary IgA concentration was significantly increased on administration of viable and heat-killed probiotic tablets. Both heat-killed and viable probiotic groups elevated salivary IgA by administrating oral tablets for 4 weeks, but without showing statistical difference to the placebo. The IgA concentration in heat-killed and viable probiotic groups showed significant increase at Week 6. However, the mechanism of IgA elevation after ceasing the probiotic administration for 2 weeks should be investigated further (Fig. 2) Braathen et al. reported similar results on the immune-modulating role of probiotic strains in the oral cavity [24]. Additionally, probiotic strains have been reported to increase fecal IgA concentration significantly in sows and piglets [25]. However, studies on IgA-alleviating symptoms in upper airway infections remain insufficient. The detailed molecular mechanism of inhibition and prevention of upper airway infections by probiotic strains CP-9, AP-32, and ET-66 could be investigated in future studies.

At present study, viable strains CP-9, AP-32, and ET-66 would elevate the level of IL-10, TFG-beta in PBMCs cells. AP-32 showed effective ability in increasing IL-10, TFG-beta in PBMCs by comparing to PHA positive control (Fig. 3a and b). It presumed that viable strains CP-9, AP-32, and ET-66 might induce B-cell secreting IL-10 and TFG-beta to increase salivary IgA level. However, the correlation between salivary IL-10, TFG-beta and salivary IgA should be tested further [26]. Besides, whether heat-killed probiotic elevated IgA via inducing IL-10 and TFG-beta are still unclear. An in vitro test indicated that heat-killed AP-32 promoted interferon-γ and IL-12p70 secretion and decreased IL-13 levels [27].

Thus, in this clinical study, the ability of probiotic CP-9, AP-32, and ET-66 strains and heat-killed AP-32 and ET-66 strains to inhibit pathogenic bacteria in the oral cavity was further validated (Fig. 4). Viable probiotic lozenges presented better anti-pathogenic ability than heat-killed lozenges via secreting organic acids, antimicrobial peptides [28], exopolysaccharides (EPSs) [29] bacteriocins [30] (Fig. 5a and b). The functional antimicrobial components produced by viable probiotic strains should be detected by high-performance liquid chromatography (HPLC) and liquid chromatography (LC)–mass spectrometry in the future [31]. It’s reported that the heat-killed probiotics possessed key advantages of viable probiotics but without the certain safety concerns of viable probiotic strains [32]. However, the antimicrobial components of heat-killed probiotics should be verified further.

## Conclusion

Mixed viable probiotic tablets, consisting of *L. salivarius* subsp. *salicinius* AP-32, *B. animalis* subsp. *lactis* CP-9, and *L. paracasei* ET-66, and heat-killed probiotics, including *L. salivarius* subsp. *salicinius* AP-32 and *L. paracasei* ET-66, exerted their beneficial functions by elevating the oral populations of *Lactobacillus* and *Bifidobacterium*, reducing *S. mutans* in the oral cavity, and increasing salivary IgA concentration in healthy participants. In vitro test results validated that viable probiotics effectively inhibited oral pathogens, including *S. mutans*, *P. gingivalis*, *F. nucleatum* subsp*. polymorphum*, and *A. actinomycetemcomitans.* Oral-nasal mucosal immunity is the frontline defense towards invading microorganisms, and this has become a crucial issue. Thus, this study will offer an alternative strategy (probiotic supplementation) to maintain oral immunity and oral health.

## Data Availability

The datasets used and/or analysed during the current study are available from the corresponding author on reasonable request.

## Code Availability

Yes.
